# Dysfunctional muscle activities and co-contraction in the lower-limb of lumbar disc herniation patients during walking

**DOI:** 10.1038/s41598-020-77150-7

**Published:** 2020-11-24

**Authors:** Wei Wang, Hui Wei, Runxiu Shi, Leitong Lin, Lechi Zhang, Shouwei Yue, Qin Zhao, Xiaofeng Jia, Ke Li, Yang Zhang

**Affiliations:** 1 Department of Physical Medicine and Rehabilitation, Qilu Hospital, Cheeloo College of Medicine, Shandong University, 107 Wenhuaxi Road, Jinan, 250012 Shandong China; 2 Laboratory of Motor Control and Rehabilitation, Institute of Biomedical Engineering, School of Control Science and Engineering, Shandong University, 17923 Jingshi Aveue, Jinan, 250061 Shandong China; 3 Department of Rehabilitation Medicine, The Affiliated Suzhou Hospital of Nanjing Medical University, Suzhou, China; 4 Department of Neurosurgery, University of Maryland School of Medicine, Baltimore, MD 21201 USA; 5 Department of Orthopedics, University of Maryland School of Medicine, Baltimore, MD 21201 USA; 6 Department of Anatomy and Neurobiology, University of Maryland School of Medicine, Baltimore, MD 21201 USA; 7 Department of Biomedical Engineering, Johns Hopkins University School of Medicine, Baltimore, MD 21205 USA; 8 Department of Anesthesiology and Critical Care Medicine, Johns Hopkins University School of Medicine, Baltimore, MD 21205 USA

**Keywords:** Neuropathic pain, Peripheral neuropathies

## Abstract

This study aimed to investigate lower-limb muscle activities in gait phases and co-contraction of one gait cycle in patients with lumbar disc herniation (LDH). This study enrolled 17 LDH patients and 17 sex- and age-matched healthy individuals. Bilateral muscle activities of the rectus femoris (RF), biceps femoris long head (BL), tibialis anterior (TA), and lateral gastrocnemius (LG) during walking were recorded. The gait cycle was divided into four phases by the heel strike and top off according to the kinematics tracks. Root mean square (RMS), mean frequency (MF), and co-contraction of surface electromyography signals were calculated. The LDH patients showed enhanced BL RMS during the single support phase (SS), second double support phase, and swing phase (SW) as well as decreased MF of RF during SS and of TA and LG during SW (*p* < 0.05). The co-contraction of the TA-LG was increased in LDH patients than in the control group (*p* < 0.05). Positive correlations were observed between TA-LG co-contraction (affected side, *r* = 0.557, *p* = 0.020; contralateral side, *r* = 0.627, *p* = 0.007) and the Oswestry disability index scores in LDH patients. LDH patients have increased BL firing rate and insufficient motor unit recruitment in specific phases in the lower limbs during walking. Dysfunction in LDH patients was associated with immoderate intermuscular co-contraction of the TA-LG during walking.

## Introduction

Lower extremity pain was frequently observed in patients with lumbar disc herniation (LDH) that caused limited motor functions, decreased locomotion capacity, and abnormal gait^1^. An abnormal gait pattern could lead to perturbation of the lower limb dynamics, increased joint stiffness, and asymmetrical loading of the lumbar spine. It was associated with inappropriate lower limb muscle activities, lower limb musculoskeletal disorders, and disorganized neural control^2^.

Some studies that focused on the paraspinal muscle observed reduced average surface electromyography (SEMG) amplitude of the lumbar multifidus during backward bending and muscle fatigue in the lower back during an isometric prone holding test in LDH patients^3,4^. However, there are fewer studies involving lower limb muscle amplitude and frequency parameters during walking in LDH patients. In a previous study, reduced frequency and altered SEMG curves (timing characteristics) of the tibialis anterior (TA) and lateral gastrocnemius (LG) were observed in the symptomatic limb of LDH patients during walking^5^. Researchers have reported delay of biceps femoris onset, decreased SEMG amplitude of the TA and medial gastrocnemius, and undifferentiated SEMG amplitude of the biceps femoris long head (BL) and vastus lateralis in patients with low back pain^6,7^. Although all lower limb muscles should be considered, previous gait studies focused on the rectus femoris (RF), biceps femoris, TA, and gastrocnemius^6,8,9^. Thus, the lower-limb muscle amplitude and frequency parameters during walking in LDH patients need to be fully explored.

Except for a particular muscle, the co-contraction between agonist and antagonist muscles, which could help understand neuromuscular system control, should also be considered during walking in LDH patients. The muscular synergistic contraction and association pattern in the lower limb played an important role during gait in healthy individuals^10,11^. A study indicated that the regulation of the co-contraction characteristics reflected an efficient adjusting mechanism for joint stability^12^. High levels of co-contraction lead to an increase in energy expenditure, fatigue, reduced physical performance, and cartilage and joint degeneration^13,14^. Older adults showed increased muscle co-contraction of the lower extremity to stiffen joints, in order to compensate for deteriorations in postural control and sensory processing^15^. A previous study showed inadequate muscular coordination of LDH patients considering the greater co-contraction between antagonistic muscles around the lumbar region during backward bending^3^. Although co-contraction reflects the neuromuscular control and coordination between muscles during walking, the lower-limb intermuscular co-contraction during walking in LDH patients has not been reported.

With the aforementioned gap in research, there is a need to extend the investigation to amplitude and frequency parameters in specific gait phases as well as co-contraction in one gait cycle of the lower limb muscles during walking in LDH patients. In view of the previous studies that hemiplegia and aging showed higher muscle co-contraction, we hypothesized that LDH patients would exhibit higher muscle co-contraction during walking, which was related to the functional level of LDH patients. In this study the effects of LDH were assessed using the amplitude and frequency parameters and co-contraction of SEMG signals from the RF, BL, TA and LG activities during walking.

## Methods

### Participants

This study enrolled 17 LDH patients and 17 healthy individuals. Each healthy participant was sex- and age-matched with one LDH patient. The sample size was determined by a power analysis. The patients were recruited from the Rehabilitation Department, Qilu Hospital, Cheeloo College of Medicine, Shandong University from July 10 to July 25, 2019. All patients had been clinically diagnosed with LDH accompanied by unilateral affected in the lower extremities by the orthopedist according to the guideline for the diagnosis and treatment of lumbar disc herniation with radiculopathy^16^. Individuals with a history of muscle injuries in their low back and lower extremities, cardiovascular and cerebrovascular diseases, cognitive difficulties, vertigo or vestibular disease, abnormal spine shape, and severe visual defects were excluded. After receiving a detailed explanation of the purpose and potential risks of the experiment, all participants provided written informed consent. The study protocols were approved by the medical ethics committee of Qilu Hospital, Cheeloo College of Medicine, Shandong University. The study was carried out in accordance with relevant guidelines and regulations.

### Experimental set-up

**Figure 1:**
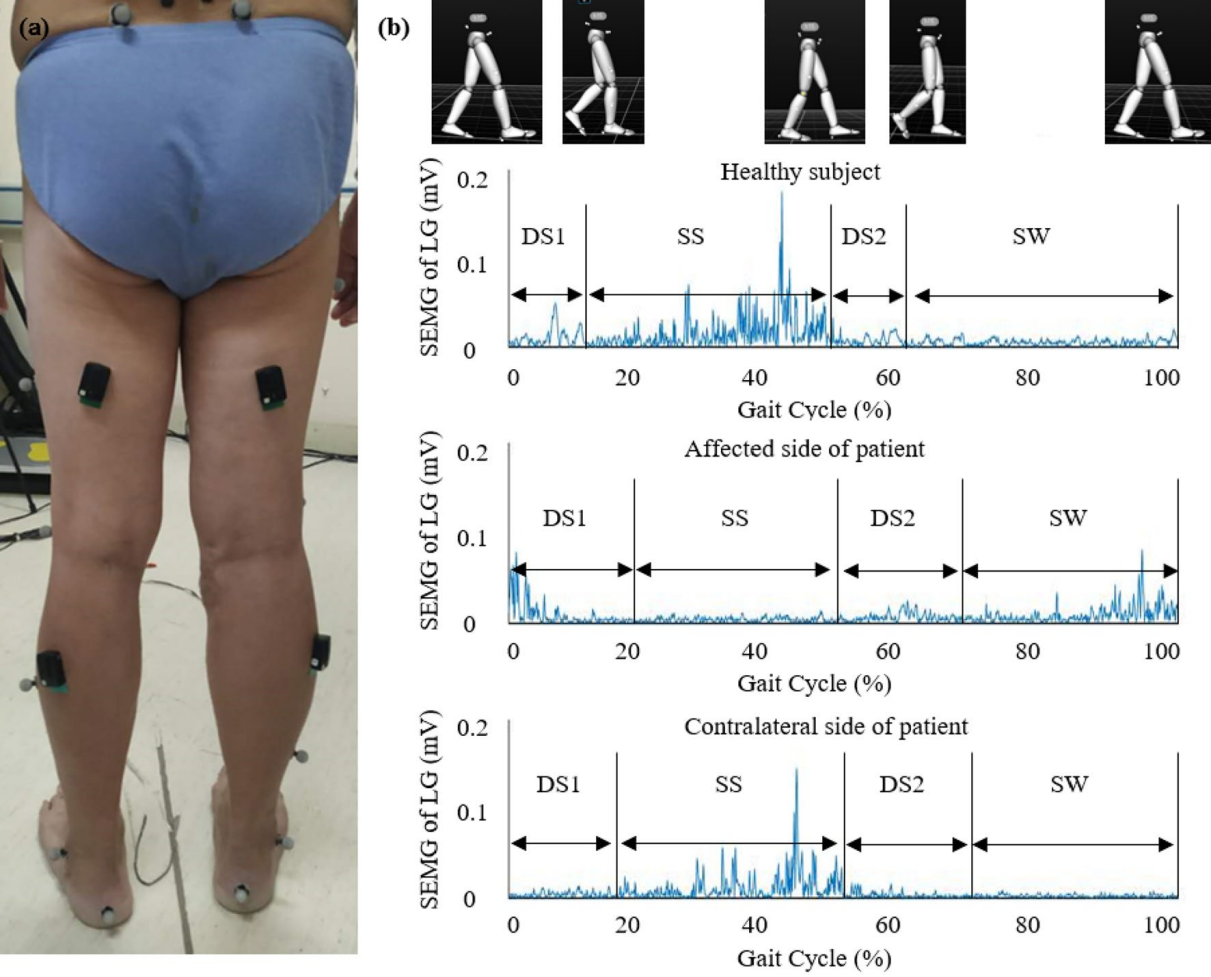
(**a**) One test participant with retro-reflective markers and surface electromyography (SEMG) electrodes in this experiment. (**b**) The full-wave rectified SEMG signals of the lateral gastrocnemius (LG) muscles during one gait cycle in one healthy participant as well as the affected and contralateral sides of a representative lumbar disc herniation (LDH) patient. (MATLAB 2016a https://www.mathworks.com).

A three-dimensional (3D) motion capture system (NaturalPoint Inc., USA) was used to obtain gait kinematics. Six cameras measured the 3D positions of 16 retro-reflective markers (Fig. 1a). The markers were affixed to the skin surface of the lower extremity. Muscle activities during walking were synchronously recorded using a wireless SEMG system (Delsys Inc., USA) (Fig. 1a). The electrodes of the RF were positioned at halfway on the line from the anterior spina iliaca superior to the superior part of the patella. The electrodes of the BL were positioned at the midpoint of the line between the ischial tuberosity and the lateral epicondyle of the tibia. Electrodes of the TA were attached on the skin surface at one-third of the line between the fibula head and the medial malleolus. Electrodes of the lateral LGs were attached on the skin surface at one-third of the line between the head of the fibula and the heel. Kinematic signals were recorded at a sampling frequency of 120 Hz, and SEMG signals were recorded at 1000 Hz.

### Test protocol

The Oswestry disability index (ODI) was used to check the functional level of LDH patients^17^. LDH patients were asked to score ten questions from 0 to 5 according to their functional ability before the walking test, which were conducted by the same rehabilitation physician. All participants were asked to walk a 3-m distance at a comfortable speed, without shoes. Before walking, the participant stood at the starting line, waiting for instructions. After hearing the instructions, he/she walked straight toward the finish line. Each participant performed four trials with a 2-min rest between trials, and they were allowed to practice once or twice to get familiarized with the testing protocol.

### Data analysis

The first gait trial was excluded, leaving three trials for each participant for the subsequent analysis. The bandpass width of the SEMG signal was 10–450 Hz, at 50 dB, and the common mode rejection ratio was < 750 mV RMS. SEMG signals were not normalized during the gait^18^. The full-wave rectified SEMG signals of the LG muscles of one healthy participant, and the affected and contralateral sides of a representative LDH patient are depicted in Fig. 1b. According to the heel strike and top off of kinematics tracks, the SEMG signals within one gait cycle were divided into the following four segments: DS1, SS, DS2, and SW. In this study, the velocity, stride time and the duration of phases of LDH patients and controls were comparable (Supplementary Table 1). The amplitudes of the SEMG signals of the RF, BL, TA, and LG were quantified using the RMS. The RMS was calculated as follows:

1 $$ RMS = \sqrt {\frac{1}{n}\sum\limits_{i = 1}^{n} {e(i)^{2} } } $$

where the *e*(*i*) is the amplitude of the SEMG signal at each time point *i*, and *n* presents the number of sampling points (i.e., the product of the sampling frequency and time length) of SEMG signals.

The frequency feature of the SEMG signals was estimated by the mean frequency (MF), which was defined as follows:

2 $$ MF = \frac{{\int_{0}^{{f_{s} /2}} {fP(f)df} }}{{\int_{0}^{{f_{s} /2}} {P(f)df} }} $$

where the *f*_*s*_ is the sampling frequency of the SEMG signal, and the *P(f)* is the power spectral density of the SEMG signal. The MF was calculated based on the Short-Time Fourier Transform (STFT). A shifting-window technique was used in the STFT, with a window width of 50 ms and shifts as long as 25 ms.

**Figure 2:**
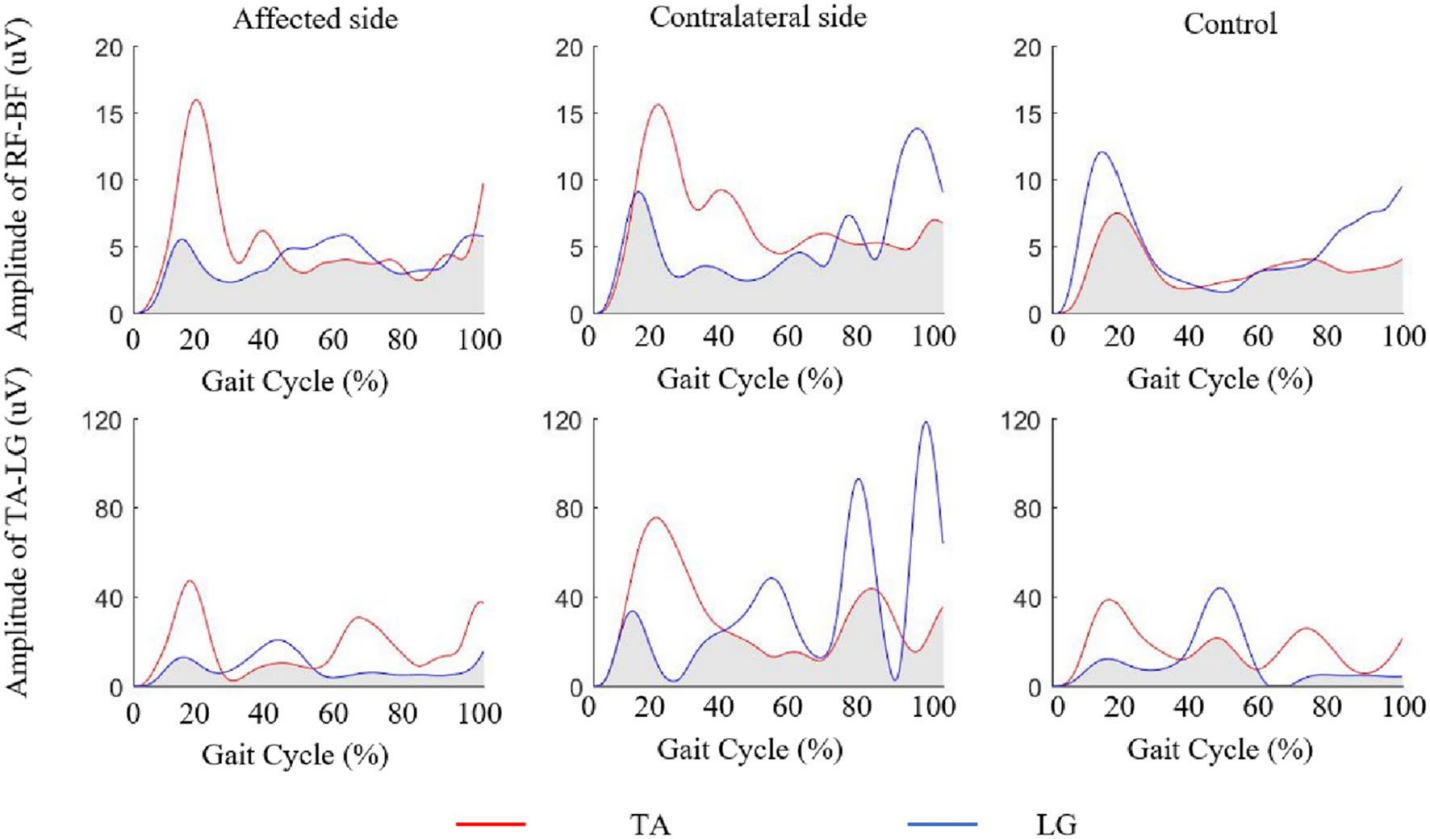
Schematic of lower limb muscle co-contraction. The representative electromyography (EMG) signals of the tibialis anterior (TA) and lateral gastrocnemius (LG) were extracted from one gait cycle in one lumbar disc herniation (LDH) patient and one healthy participant. The common area is the overlapping region under the curves of the EMG signals and represents the amount of muscle co-contraction. (MATLAB 2016a https://www.mathworks.com).

Muscle co-contraction was defined as the common activity presented as a percentage of total muscle activity in a pair of antagonist muscles within a gait cycle^19^. Figure 2 showed the schematic of lower limb muscle co-contraction.

3 $$ CC = \frac{{\int_{0}^{n} {{\text{[e}}_{1} (i) + e_{2} (i)]di} - \int_{0}^{n} {{\text{|e}}_{1} (i) - e_{2} (i)|di} }}{{\int_{0}^{n} {{\text{[e}}_{1} (i) + e_{2} (i)]di} }}{\text{*100\% }} $$

where the *e*_*1*_(*i*) and *e*_*2*_(*i*) are the amplitudes of a pair of SEMG signals at each time point *i*, and *n* presents the length of SEMG signals*.* The parameters were calculated using MATLAB 2016a (MathWorks, Natick, MA, USA).

**Table 1:** Descriptive characteristics of subjects.

Number	LDH patients (n = 17)						Control (n = 17)			
	Gender	Age (years)	BMI	Height (cm)	Affected side	ODI	Gender	Age (years)	BMI	Height (cm)
1	M	43	26.03	172	L	19	M	40	23.84	175
2	M	45	30.74	173	R	26	M	43	22.84	180
3	M	65	30.45	170	R	28	M	67	25.46	188
4	M	47	27.18	165	L	29	M	45	22.22	180
5	M	53	27.72	172	R	25	M	54	23.53	170
6	F	39	20.81	155	R	10	F	40	19.05	162
7	F	44	19.53	160	R	24	F	44	21.08	163
8	F	56	24.54	164	L	28	F	55	26.84	158
9	M	57	24.22	170	L	16	M	60	28.34	168
10	M	38	28.30	155	R	11	M	39	23.12	174
11	F	45	20.83	161	L	16	F	48	20.34	156
12	F	56	21.56	164	R	17	F	57	28.52	160
13	M	52	24.61	165	R	33	M	53	30.39	174
14	F	53	28.65	165	R	21	F	52	27.34	153
15	F	55	22.86	162	L	8	F	57	21.02	155
16	F	66	27.06	155	R	33	F	68	27.12	156
17	M	49	22.86	175	L	24	M	46	23.66	172

*LDH* lumbar disc herniation,* n* number of subjects,* BMI* body mass index,* ODI* oswestry disability index.

Statistical analyses were performed using SPSS 20.0 (SPSS Inc., Chicago, IL). The Kolmogorov–Smirnov test showed all variables with a normal distribution. All numerical data are shown as means ± standard error of the mean (SEM). Data were compared across the following three groups: (1) control group, (2) affected side of LDH patients, and (3) contralateral side of LDH patients. For healthy individuals, only one side (corresponding to the affected side of the LDH patient) was selected. An independent *t*-test was applied to examine the differences between LDH patients (both affected and contralateral sides) and the control group. A paired *t*-test was used to examine the difference between the affected side and the contralateral side. No interaction was considered across these *t*-tests. Similarly, bivariate correlation analysis was performed to assess the correlations between the ODI and magnitude, frequency, and co-contraction features of muscles during walking for the affected and contralateral sides. The values of *p* < 0.05 were considered statistically significant.

## Results

**Figure 3:**
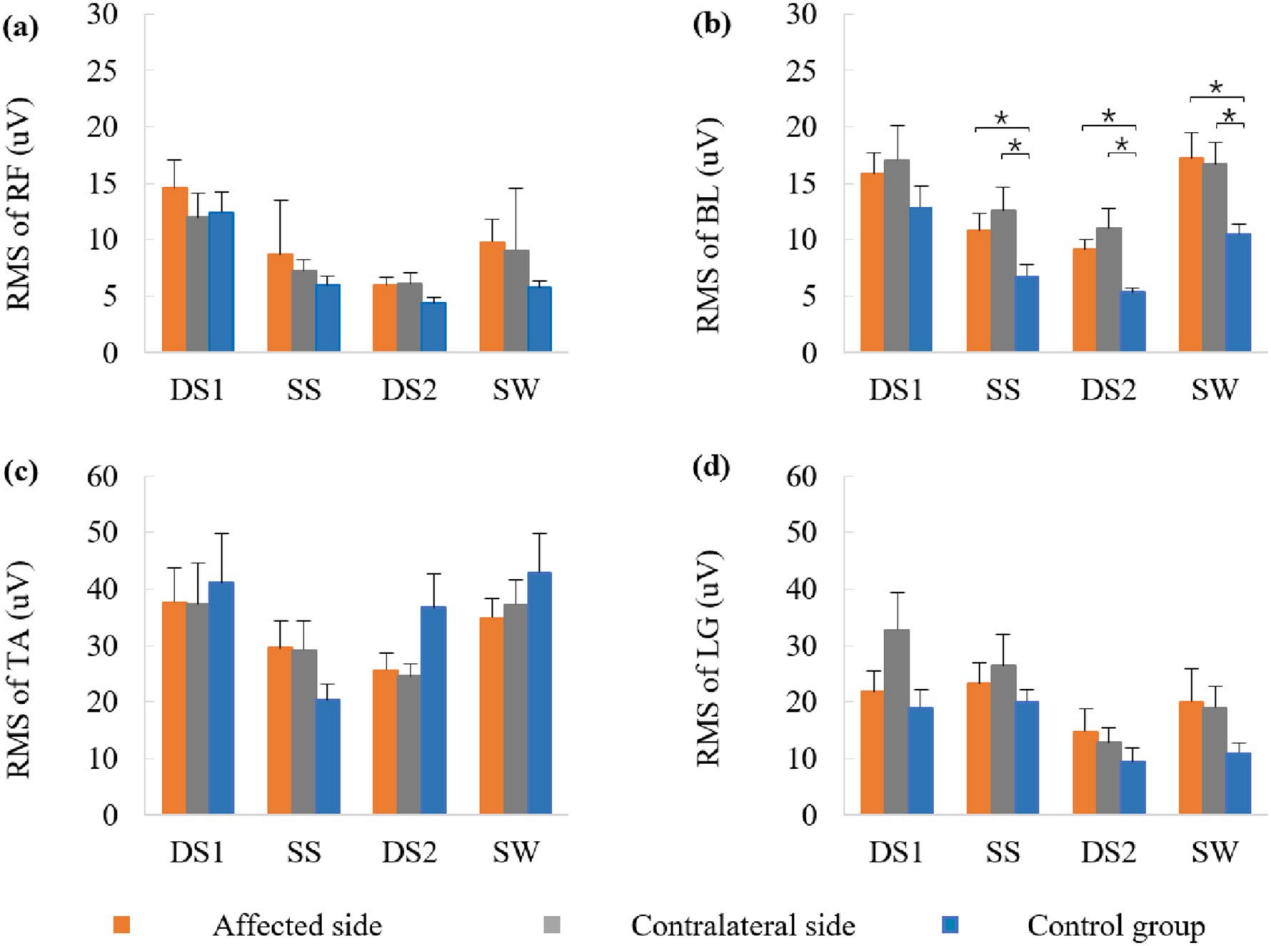
The surface electromyography (SEMG) amplitude in (**a**) rectus femoris (RF), (**b**) biceps femoris long head (BL), (**c**) tibialis anterior (TA) and (**d**) lateral gastrocnemius (LG) muscles during the first double stance phase (DS1), single stance phase (SS), the second double stance phase (DS2), and swing phase (SW) of the affected side, contralateral side, and control.

The characteristics of 17 LDH patients (8 women and 9 men; aged 50.76 ± 1.91 years) and 17 healthy individuals (aged 51.06 ± 2.12 years) are shown in Table 1. There is no significant difference in BMI (*p* = 0.503) and height (*p* = 0.423) between LDH and control groups. Results of the RF, BL, TA, and LG contraction magnitudes during DS1, SS, DS2, and SW of the gait cycle are shown in Fig. 3. The BL of the affected side and contralateral side showed significantly higher RMS values in SS (affected side: *t* = 2.067, *p* = 0.048; contralateral side: *t* = 2.468, *p* = 0.021), DS2 (affected side: *t* = 3.642, *p* = 0.001; contralateral side: *t* = 2.996, *p* = 0.008) and SW (affected side: *t* = 2.579, *p* = 0.018; contralateral side: *t* = 2.754, *p* = 0.011) than that in healthy controls. No significant difference was observed in the RMS values of the RF, TA and LG muscles between the affected and contralateral sides, between the affected side and controls, or between the contralateral side and controls (*p* > 0.05, Fig. 3).

**Figure 4:**
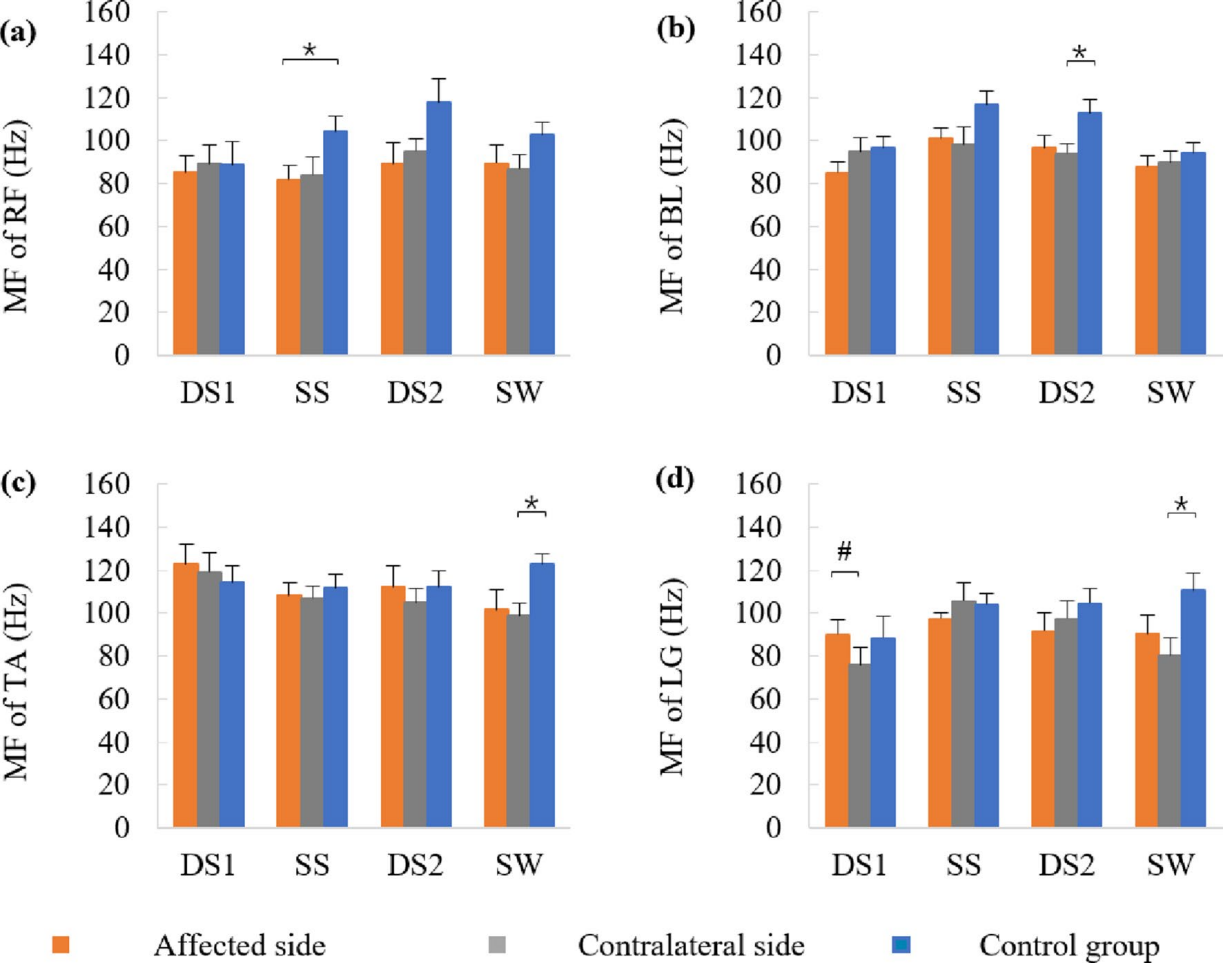
The mean frequency (MF) of surface electromyography (SEMG) in (**a**) rectus femoris (RF), (**b**) biceps femoris long head (BL), (**c**) tibialis anterior (TA), and (**d**) lateral gastrocnemius (LG) muscles during the first double stance phase (DS1), single stance phase (SS), the second double stance phase (DS2) and swing phase (SW) of the affected side, contralateral side, and control.

The frequency parameters of the RF, BL, TA, and LG activities during DS1, SS, DS2, and SW of the gait cycle are displayed in Fig. 4. Decreases in the MF of the RF during the SS of the gait cycle were observed in the affected side compared with the control group (*t* = − 2.224, *p* = 0.033). Significant differences were observed between the contralateral side and the control groups in terms of MF values of the BL during DS2 (*t* = − 2.206, *p* = 0.036), TA during SW (*t* =  − 3.001, *p* = 0.005), as well as LG during SW (*t* = − 2.588, *p* = 0.014) of the gait cycle. The MF of the LG in the affected side was higher than that of the contralateral side during DS1 of the gait cycle (*t* = 2.180, *p* = 0.045, Fig. 4).

**Figure 5:**
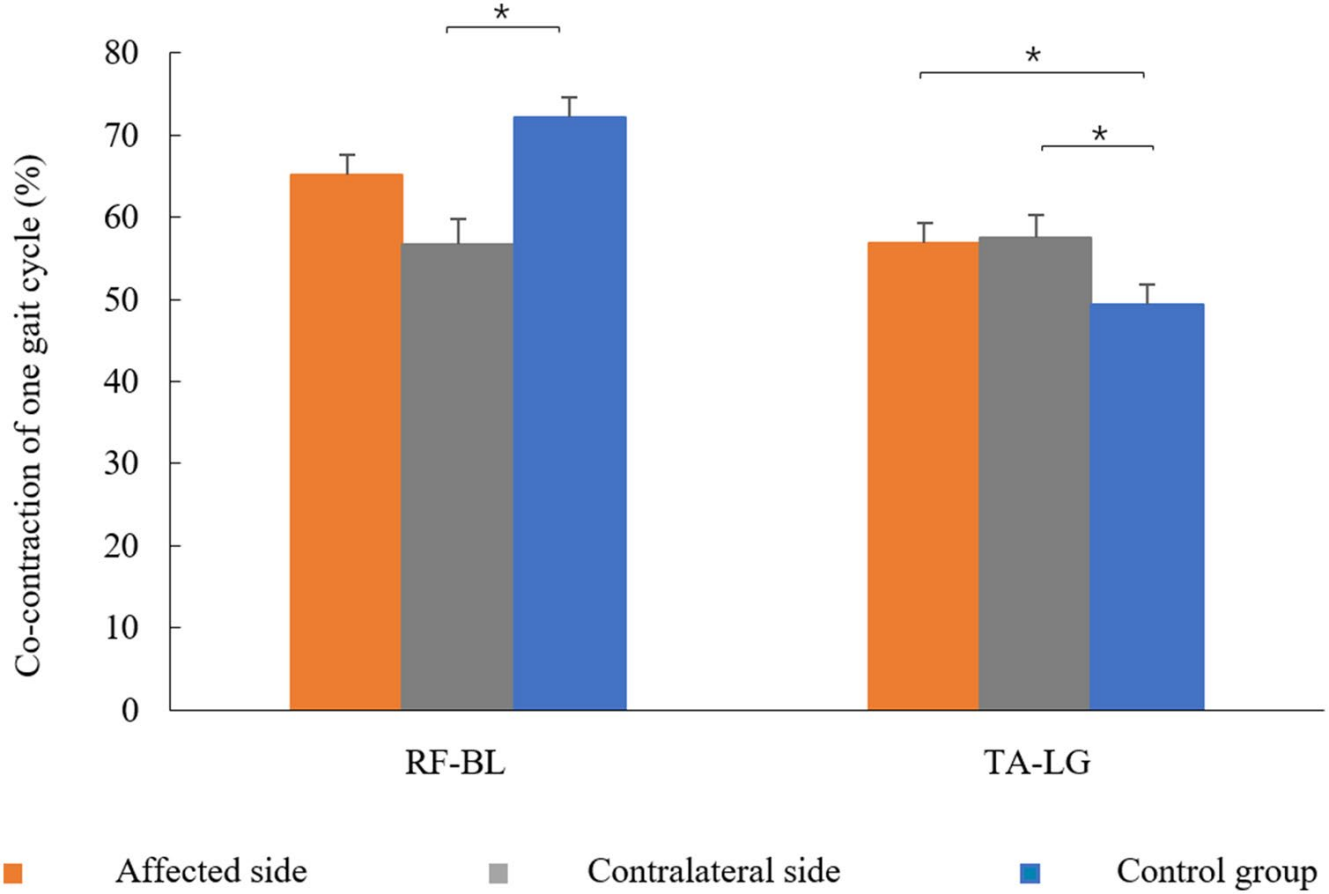
The co-contraction between rectus femoris (RF) and biceps femoris long head (BL), as well as tibialis anterior (TA) and lateral gastrocnemius (LG) of one gait cycle in the control and the affected and contralateral sides.

Muscular co-contractions between the TA and LG, as well as the RF and BL in one gait cycle, are shown in Fig. 5. The RF-BL co-contraction in the contralateral side was significantly lower than those in the control group (*t* = −3.953, *p* = 0.000). TA-LG co-contraction was increased in LDH patients (affected side: *t* = 2.337, *p* = 0.026; contralateral side: *t* = 2.151, *p* = 0.039) compared with the control group. No significant difference was observed in RF-BL co-contraction between the affected side and the control groups or in TA-LG co-contraction between the affected side and contralateral side (*p* > 0.05).

**Figure 6:**
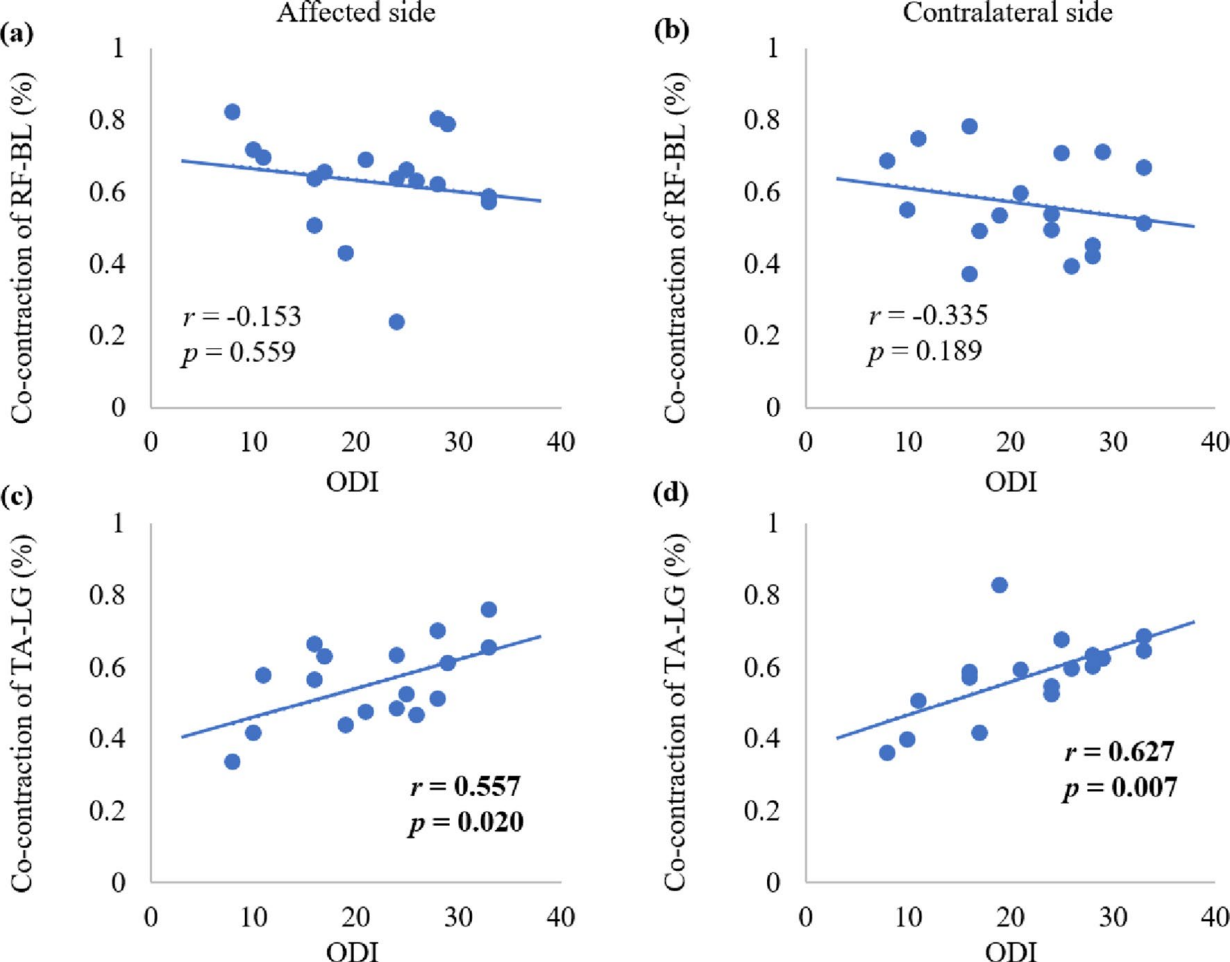
The relationship between muscle co-contraction and Oswestry disability index (ODI) scores in lumbar disc herniation (LDH) patients.

The relationships between muscular co-contractions of one gait cycle and the ODI scores are shown in Fig. 6. The TA-LG co-contraction in the affected side (*r* = 0.557, *p* = 0.020) and the contralateral side (*r* = 0.627, *p* = 0.007) were positively correlated with the ODI scores. No significant correlation was observed between the ODI scores and co-contraction of the RF-BL in the affected side (*r* = -0.153, *p* = 0.559) or contralateral side (*r* = − 0.335, *p* = 0.189).

## Discussion

This study investigated the muscle activities and intermuscular co-contraction in the affected and contralateral sides of LDH patients and healthy controls (sex- and age-matched) during walking. The SEMG amplitude, frequency, and co-contraction features in lower-limb muscles were assessed. Similarly, the bivariate correlations between muscle co-contraction and ODI were assessed in LDH patients. The spatiotemporal gait feature, such as the velocity, stride time and the duration of phases were comparable in LDH patients and controls. Decreased SEMG MF at specific phases of the gait cycle in LDH patients indicated insufficient recruitment of motor units and muscle fatigue. The results suggested that LDH patients have enhanced BL contraction intensity, muscle fatigue in specific phases, and immoderate muscle co-contraction in the lower limbs during walking. This study can help us determine the best-targeted strategies for the BL activity and TA-LG coordination and find an objective evaluation index for LDH patients in clinical settings.

The amplitude of SEMG reflects the motor unit discharge and has been considered an index of muscle contraction intensity. Farahpour et al. reported comparable activity magnitude of BL during the stance phase among low back pain patients with pronated feet and controls^7^. However, a study found a prolonged onset time of biceps femoris during the SW of the gait cycle in patients with low back pain^6^. In this study, the BL in both affected and contralateral sides of LDH patients showed significantly higher SEMG magnitude in SS, DS2, and SW phases of the gait cycle than in those of healthy controls. The increased BL contraction intensity of patients with LDH revealed greater muscle firing rate to ensure support by knee flexion during the stance phase and prolonged onset during the swing phase^6,20^. In clinical practice, weakening the BL activity of LDH patients may facilitate to gait function rehabilitation. No significant difference was observed in the amplitudes of the RF, TA, and LG, which suggested that these muscles can contribute to appropriate contraction intensity in each phase during walking, or the contraction pattern was inconsistent among patients during phases of the gait cycle.

Although no significant difference was observed in the amplitudes of the RF, TA, and LG. In the frequency domain, the RF, TA, and LG in LDH patients were observed to have significantly lower MF in the SS and SW phases. The spectral frequency of SEMG correlates with the average conduction velocity and has been used to evaluate type I and II muscle fiber activations and the recruitment of motor units during muscle contraction^21^. The SEMG MF of LDH patients was reduced, indicating inadequate recruitment of motor units and muscle fatigue in specific gait phases during walking^22^. The lowered MF values of the BL during DS2 and LG during SW generated less than necessary muscular activation for propulsion and knee flexion in the contralateral side of LDH patients^23^. In LDH patients, the TA showed decreased MF in the SW phase of the gait cycle, which may impair ankle dorsal flexion and foot clearance during walking^24^. In the SS of the gait cycle, the frequency of the RF in affected side of LDH patients declined, which may indicate insufficient conduction velocity and recruitment of motor units for the support phase. These abnormal muscular activities could result from the alteration of the gait kinematics pattern—reduce the contribution of the affected leg and rely on the contralateral leg to relieve pain during walking or the truck performance^25^. LDH patients should strengthen muscle activities of the TA and LG, especially the RF, and weaken the muscle activity of the BL by choosing an appropriate rehabilitation training.

Our findings are consistent with those of previous studies showing greater co-contraction between antagonistic muscles around the lumbar region of LDH patients and in the lower limb of older adults; additionally, this observation is extended to the lower limb muscles of LDH patients during walking^3,19^. The TA-LG co-contraction increased in LDH patients as an inefficient muscular coordination strategy, which can compensate for pain, numbness, stability, and proprioception caused by prolonged muscle co-contraction or disordered timing of muscle activation in the TA and LG during walking^5,26^. The decreased RF-BL co-contraction in the contralateral side of LDH patients compared with the healthy group could be associated with the increased amplitude of BL activities. Inefficient intermuscular coordination may lead to greater energy expenditure, insufficient toe clearance after toe-off, muscle fatigue, and further impairment of physical function during walking^26,27^. In clinical rehabilitation training, the selectivity in movement control, such as voluntary movement and neuromuscular activation, should be considered in LDH patients with inappropriate co-contraction during walking^28^.

Nader and Hunt et al. reported about the relationships between muscular activities and the kinematic index during the stance phase of the gait cycle in LDH patients and healthy individuals^7,24^. However, the correlations between muscular co-contraction and dyskinesia during walking remain unknown. Our study showed the correlations between muscle TA-LG co-contractions during walking and the ODI scores in the affected and contralateral sides of LDH patients, which implied that the dysfunction of LDH patients was affected by lower limb muscular coordination^29^. These findings suggest that patients with high ODI scores experience excessive synchronous contraction of the lower limbs. Muscle co-contraction, which may partially explain the mobility impairments associated with LDH. The co-contraction can be used as an alternative evaluation indicator for rehabilitation training, which is sensitive and objective. Therefore, targeted treatment should be considered in clinical practice. Future studies are needed to identify whether muscle co-contraction is typically a reactive or an anticipatory response.

This study has some limitations. This study has a retrospective design and analyzed 17 LDH patients from a single center. In addition, patients were diagnosed with multiple-segment intervertebral disc herniation and only a 3-m distance was provided for walking test. Finally, only the RMS, MF, and co-contraction algorithms were applied in the analysis of SEMG signals to determine the pathological biomarkers of muscle activities in LDH patients.

In conclusion, LDH patients experience increased BL firing rate, insufficient motor unit recruitment in specific phases, and excessive muscle co-contraction in the lower limbs during walking. Dysfunction (ODI scores) was positively correlated with intermuscular co-contraction of the TA-LG on both sides of LDH patients. This abnormal muscular coordination pattern could lead to excessive energy expenditure, muscle fatigue, and further impairment of physical function during walking.

## Supplementary information

Supplementary Data 1.

Supplementary Table 1.
