# Supplementary material for: Dysfunctional muscle activities and co-contraction in the lower-limb of lumbar disc herniation patients during walking
Source: Sci Rep. 2020 Nov 24;10:20432. doi: 10.1038/s41598-020-77150-7 (PMC7686363; doi:10.1038/s41598-020-77150-7)
Supplement: Supplementary file 2 — Supplementary Table 1. [file 41598_2020_77150_MOESM2_ESM.docx]

Supplementary table 1. Spatiotemporal gait feature.

| **Number** | **Affected side of LDH patients** | | | | | | **Contralateral side of LDH patients** | | | | | | **Controls** | | | | | |
| --- | --- | --- | --- | --- | --- | --- | --- | --- | --- | --- | --- | --- | --- | --- | --- | --- | --- | --- |
|  | Velocity (m/s) | Stride time (s) | DS1 (%) | SS (%) | DS2 (%) | SW (%) | Velocity (m/s) | Stride time (s) | DS1 (%) | SS (%) | DS2 (%) | SW (%) | Velocity (m/s) | Stride time (s) | DS1 (%) | SS (%) | DS2 (%) | SW (%) |
| 1 | 0.42 | 1.63 | 11 | 34 | 17 | 38 | 0.42 | 1.58 | 18 | 39 | 11 | 32 | 0.68 | 1.32 | 15 | 33 | 15 | 37 |
| 2 | 0.39 | 1.55 | 23 | 28 | 20 | 30 | 0.42 | 1.54 | 20 | 30 | 23 | 28 | 0.52 | 1.67 | 16 | 30 | 16 | 39 |
| 3 | 0.43 | 1.53 | 17 | 33 | 19 | 30 | 0.44 | 1.63 | 21 | 32 | 19 | 28 | 0.53 | 1.29 | 8 | 42 | 8 | 43 |
| 4 | 0.52 | 1.38 | 20 | 31 | 15 | 34 | 0.48 | 1.27 | 16 | 28 | 22 | 34 | 0.55 | 1.38 | 16 | 36 | 16 | 32 |
| 5 | 0.53 | 1.70 | 12 | 41 | 10 | 38 | 0.43 | 1.67 | 10 | 39 | 11 | 40 | 0.64 | 1.41 | 11 | 36 | 15 | 39 |
| 6 | 0.79 | 1.22 | 8 | 39 | 13 | 41 | 0.76 | 1.17 | 12 | 43 | 8 | 37 | 0.99 | 1.09 | 12 | 40 | 6 | 43 |
| 7 | 0.54 | 1.16 | 14 | 37 | 15 | 34 | 0.62 | 1.18 | 15 | 35 | 14 | 36 | 0.63 | 1.37 | 13 | 37 | 12 | 38 |
| 8 | 0.77 | 1.29 | 14 | 39 | 8 | 39 | 0.75 | 1.32 | 8 | 40 | 14 | 39 | 0.63 | 1.13 | 11 | 40 | 13 | 36 |
| 9 | 0.78 | 1.13 | 13 | 39 | 10 | 38 | 0.87 | 1.17 | 10 | 39 | 13 | 38 | 0.61 | 1.58 | 10 | 42 | 15 | 33 |
| 10 | 0.63 | 1.33 | 16 | 31 | 15 | 39 | 0.61 | 1.39 | 16 | 36 | 16 | 32 | 0.63 | 1.63 | 14 | 37 | 17 | 32 |
| 11 | 0.54 | 1.13 | 19 | 32 | 13 | 36 | 0.50 | 1.08 | 13 | 38 | 20 | 29 | 0.53 | 1.28 | 10 | 47 | 6 | 37 |
| 12 | 0.75 | 1.13 | 15 | 38 | 14 | 33 | 0.72 | 1.20 | 15 | 35 | 15 | 35 | 0.70 | 1.23 | 14 | 34 | 12 | 40 |
| 13 | 0.77 | 1.40 | 9 | 36 | 14 | 41 | 0.79 | 1.34 | 13 | 43 | 9 | 35 | 0.61 | 1.38 | 14 | 30 | 16 | 39 |
| 14 | 0.55 | 1.27 | 15 | 33 | 15 | 37 | 0.55 | 1.23 | 14 | 39 | 14 | 32 | 0.60 | 1.09 | 15 | 35 | 12 | 38 |
| 15 | 0.53 | 1.48 | 16 | 34 | 16 | 34 | 0.54 | 1.49 | 16 | 35 | 16 | 34 | 0.54 | 1.62 | 16 | 33 | 18 | 33 |
| 16 | 0.49 | 1.44 | 13 | 34 | 20 | 33 | 0.42 | 1.56 | 22 | 27 | 14 | 37 | 0.43 | 1.52 | 17 | 34 | 17 | 31 |
| 17 | 0.60 | 1.25 | 7 | 41 | 13 | 40 | 0.61 | 1.24 | 13 | 40 | 7 | 41 | 0.95 | 1.25 | 10 | 39 | 11 | 40 |
